# Folie à deux ou le partage de symptômes comme modalité relationnelle: à propos d’un cas

**DOI:** 10.11604/pamj.2019.32.47.8378

**Published:** 2019-01-28

**Authors:** Amine Bout, Nabil Berhili, Hayat Hlal, Rachid Aalouane, Ismail Rammouz

**Affiliations:** 1 Service de Psychiatrie, Centre Hospitalier Universitaire Hassan II, Fès, Maroc

**Keywords:** Folie à deux, trouble psychotique partagé, délire hystérique, Folie à deux, shared psychotic disorder, hysterical delirium

## Abstract

Le trouble psychotique partagé ou Folie à deux (FD) est une entité rare et controversée qui pose des problèmes d’ordre phénoménologique, nosographique et psychopathologique. Elle interroge la nature du délire et sa survenue en dehors d’une structure psychotique ainsi que la question de la contagion du symptôme en psychiatrie. Nous proposons l’analyse sémiologique d’un cas intrafamilial de Folie à deux, cas dont la particularité clinique est le partage de symptômes délirants mais aussi d’éléments psychotiques non délirant (déficitaires). A travers quoi nous revenons sur les caractéristiques épidémiologiques et les facteurs communs avec les autres cas rapportés dans la littérature dans différentes cultures. Nous dressons ensuite l’historique de l’entité et son évolution au fil des classifications depuis sa première description par Legrand jusqu’au DSM 5. Et de là, nous soulignons le caractère insuffisant d’une approche purement descriptive et mettons l’accent sur le lien possible avec d’autres situations cliniques plus fréquentes avec comme point commun la transmission entre deux ou plusieurs personnes de symptômes psychiques. Puis, nous proposons une réflexion psychopathologique qui s’axe essentiellement sur le partage du symptôme et non sa nature tout en interrogeant la fonction du délire au sein d’un couple délirant.

## Abstract (en)

Shared Psychotic Disorder or Folie à deux is a rare and controversial entity that raises phenomenological, nosographic and psychopathological issues. It questions the nature of dilusion and its occurrence outside the psychotic structure and also the issue of symptoms contagion in psychiatry. We offer a semiological analysis of an intrafamilial case of Folie à deux, a case with the clinical specificity of sharing not only delusional symptoms but also non-delusional psychotic elements. We then dig back in the epidemiological characteristics and common factors to other reported cases in the literature of different cultures. Then we draw a history of the entity and its evolution over the classifications since its first description by Legrand to the DSM 5. From here, we emphasize the insufficiency of a purely descriptive approach and focus on the possible link with other more common clinical situations of mental symptoms transmission between two or more persons. Then, we propose a psychopathological reflection that essentially targets the sharing of the symptom rather than its nature while questioning the function of the delusion in a delusional couple.

## Introduction

Le trouble psychotique partagé est une entité psychiatrique rare décrite en premier par Legrand et baptisée "Folie à deux". Il désigne une situation clinique ou à la faveur d’un confinement particulier au contact d’un sujet délirant, un deuxième sujet adopte ce même contenu idéique délirant. Cette éventuelle diffusion du délire d’un sujet à l’autre reste intrigante et interroge la nature même du délire et de la maladie mentale. Ce trouble à toujours suscité un intérêt particulier malgré son caractère fuyant difficile à définir, et l’une des entraves majeure à sa compréhension est sa rareté, et donc le peu d’éléments épidémiologiques disponibles qui restent confinées aux cas rapportés dans la littérature. Ce qui fait qu’à ce jour, peu d’avancées ont été faites dans le sens de l’élucidation des mécanismes psychopathologiques en jeu et dans la définition d’un cadre nosographique pour cette entité qui demeure confuse.

## Patient et observation

L’incidence du trouble serait estimée entre 1,7 et 2,6% des cas d’hospitalisation en psychiatrie [1]. Ces cas sont majoritairement issus de la même famille avec prédominance de duos de sexe féminin, et le cas de deux sœurs étant le plus fréquent en occident [2]. Dans une étude qui rapporte 64 cas recensés de 1993 à 2005 [3], la nature de la relation est distribuée comme suit: 97.6% de cas de parenté au premier degré; 2.4% entre des amis sans lien génétique; et 52.4% au sein de couples mariés. Albeit et al trouve que 50% des cas sont des couples de mères-filles ou de sœurs [4]. En Asie, une étude japonaise a regroupé 97 cas sur une durée de 90 ans. Il retrouve une prédominance de transmission de mères à enfant et au sein de couples mariés [5]. Un isolement social est retrouvé dans 64 à 84% des cas [3], la durée de la relation entre les deux protagonistes est très variable mais souvent au-delà de plusieurs semaines avec une moyenne de 72 semaines [3]. Le diagnostic le plus retrouvé parmi les cas rapportés chez le sujet dit inducteur est le trouble délirant (délire de persécution et délire mégalomaniaque) suivi par la schizophrénie puis les troubles de l’humeur. Sur 72 cas rapportés entre 1942 et 1993, et chez les cas secondaires, 89% présentaient une comorbidité psychiatrique type dépression, démence ou retard mental.

**De Legrand au DSM 5:** Le concept de FD apparait pour la première fois en 1871 avec Legrand du Saulle [6] qui le décrit dans son ouvrage « le délire des persécutions ». Il parle d’un personnage actif, influent et un personnage passif qui subit les idées délirantes. En 1877, Lasègue et Falret publient l’article intitulé: Folie à deux [7], la présentant comme une entité nosographique à part et la dotant d’une description sémiologique précise et surtout proposent des condition à cette transmission dont les plus importantes sont: d’abord que le sujet réceptif soit d’intelligence faible et plus enclin à la passivité qu’à l’émancipation, puisque les deux sujets vivent en relation constante; ensuite que le délire soit vraisemblable. Il présente également comme condition importante que le sujet passif ait un intérêt particulier à partager le symptôme, ainsi il exprime cette notion de bénéfice qu’il exprime ainsi: ***« on ne cède pas à la pression de la folie que si elle vous fait entrevoir la réalisation d’un rêve caressé ».***

Régis propose de scinder cette entité en folie communiqué, celle-ci correspondant à la description de Lasègue et en folie simultané correspondant à la déclaration simultanée d’une psychose chez deux sujets prédisposés. En 1881, Marandon de Montyel [8] rajoute une nouvelle dérivation de FD et change la dénomination. Il isole alors: folie imposée correspondant à la description de Lasègue et Falret; la folie simultanée de Régis et une troisième, la folie communiquée dans laquelle un aliéné communique ses hallucinations et ses conceptions délirantes à un autre individu, héréditairement prédisposé. Clérambault fut parmi les premiers à mettre au point la distinction entre folie et délire. Pour lui ce qui se transmet se sont les thèmes idéiques et une partie des fonds affectifs, pas la folie en tant que telle, et encore moins ce qui il appelle les mécanismes générateurs de cette folie. Les psychanalystes avec Lacan parlent d’interaction inconsciente dépassant le cadre du simple empreint de symptômes. Et souligne la place du degré de transfert établi entre les deux patients. Les classifications modernes adoptent la dénomination de trouble psychotique partagé aussi bien dans la CIM 10 que dans le DSM IV. La définition de cette entité est basé sur la notion de mise en place d'un système délirant, consécutivement à une relation étroite avec un sujet dit primaire ou inducteur, celui-là est déjà porteur d’un trouble avéré ce qui signifie que le diagnostic de trouble délirant partagé ne tombe que sur le sujet dit induit. Un contenu délirant similaire est requis et un critère d’élimination écartant le diagnostic en cas de trouble de l’humeur ou d’un autre trouble de nature psychotique. Cette définition reste fidèle aux descriptions classiques, cependant elle limite le trouble psychotique au délire et fait fi de la possibilité de partage d’autres symptômes de nature psychotique. L’avènement du DSM 5 confirme cette tendance réductionniste et confond le trouble psychotique partagé tel qu’il a été défini classiquement mais aussi dans le DSM IV- avec le trouble délirant. En effet, en présence de critères d’inclusion du trouble délirant le diagnostic de trouble délirant est posé; ce n’est qu’en l’absence de la totalité des critères que cette entité est renvoyée vers le chapitre: autres troubles du spectre schizophrénique et autres troubles délirants sous le nom de symptômes délirants chez un partenaire d’individu avec trouble délirant et ici symptôme délirant désigne une simple croyance dans le délire d’un individu dominant et délirant. L’éventualité que le sujet primaire soit schizophrène ou porteur d’un autre diagnostic n’est pas évoquée. Le trouble psychotique partagé s’est retrouvé donc écarté de la classification car le fait de le considérer comme un trouble délirant à part entière néglige cette notion de partage du symptôme au sein d’une relation intime, et d’un autre coté le réduire à une simple crédulité ne rend pas compte de l’entité comme elle est décrite classiquement. La question du cadre nosographique reste toujours d’actualité ce qui se traduit par la difficulté d’apporter des critères objectifs à un concept aux contours confus. Cette difficulté résulte également de l’absence de preuves psychopathologiques fiables.

### Le cas clinique

Deux sœurs H et K, âgées respectivement de 38 et 42 ans, se barricadèrent dans un étage de la maison familiale depuis 4 mois et coupèrent tout contact avec leurs parents, réagissant par une grande hostilité à chaque fois que la famille essaye d’entrer en contact avec elles. La réclusion se prolongea et se compliqua par un comportement de collectionnisme et d’accumulation de déchets, le couple se négligea sur le plan vestimentaire et sombra dans une incurie totale. Apres une évolution de 7 mois la famille consentit enfin à faire intervenir les autorités pour les déloger et les cheminer pour une prise en charge hospitalière. Le premier contact avec H et K a eu lieu aux urgences psychiatriques, elles se présentèrent dans un état d’agitation extrême criant de concert les même propos injurieux envers leur père, utilisent les même phrases et les même tournures. Physiquement hors mis leur état d’incurie manifeste, on a noté une perte importante de poids chez les deux sœurs plus marquée pour K. Elles sont d’une famille modeste et nombreuse. K est l’ainée de la fratrie. Les deux n’ont pas été scolarisées et ont travaillé très tôt comme tisserandes. K. Est décrite comme protectrice et autoritaire avec ses sœurs. Elle se comporte avec elles en chef d’entreprise, gère leur argent étant la seule à posséder un compte bancaire. Elle est travailleuse sérieuse, exigeante. H est la cadette de la famille, Le caractère fusionnel de la relation entre les deux sœurs a été souligné par tous les membres de la famille. H considérait K comme une deuxième mère, celle-ci ayant de l’autorité et de l’influence sur elle. H est décrite comme immature et suggestible, elle dépend beaucoup de de K. La famille rapporte aussi que la première à avoir manifesté une hostilité envers le père et des accusations d’ensorcellement était bien la sœur ainée K. Elle se mit à vérifier les fonds de verres et à préparer elle-même ses repas. Elle devint rapidement inerte, en retrait et progressivement incurique, une évolution insidieuse qui s’étala sur 9 mois. La patiente H fut la seule à garder une proximité avec K tout en gardant ses distances par rapport au comportement délirant de sa sœur. K se réfugia dans un étage de la maison familiale et H la suivit après 3 mois dans sa retraite. La persécution centrée au départ sur le père s’est ensuite étendue aux autres membres de la famille. Durant 7 mois, les deux sœurs s’étaient nourries de restes d’aliments retrouvés dans les poubelles. Elles devinrent très incuriques, accumulèrent des déchets et des objets métalliques à leur étage. Cliniquement, après hospitalisation, la patiente K présente un délire de persécution et d’ensorcellement diffus mal systématisé à mécanisme essentiellement intuitif avec conviction inébranlable, une rationalisation excessive du comportement et une banalisation de la situation, une neutralité de l’humeur qui tend vers un certain détachement et un émoussement de l’affectivité ainsi quelques troubles de raisonnement type rationalisme morbide, un déficit cognitifs avec défauts d’abstractions. Un bilan organique est revenu normal, et le diagnostic d’une schizophrénie tardive type indifférenciée a été posé. H. a présenté les mêmes idées délirantes de persécution (des personnes tenteraient de les tuer elle et sa sœur en enrôlant les membres de leur famille), elle se montre réticente et refuse de détailler le contenu délirant, gardant une certaine méfiance vis à vis des soignants. Elle ne donne aucune explication quant aux troubles de comportement, avec un mutisme sélectif quant à certaines questions. On a noté l’absence de symptômes dissociatifs intellectuels ou affectifs. H demandait sans cesse à rester auprès de sa sœur et refuse de s’en éloigner menaçant après une première tentative de séparation de passer à l’acte suicidaire à deux. Un traitement à base de Respiridone à été instauré pour la patiente K et la patiente H a été maintenue uniquement sous un traitement anxiolytique. Une séparation totale n’a pas pu être réalisée et les deux sœurs ont continué à se voir en présence d’un soignant. A 15 jours de l’admission on note une amélioration chez la patiente H. elle a commencé à mieux coopérer et à prendre contact avec l’équipe soignante loin des conceptions délirantes sur lesquelles elle ne veut plus revenir. Un lien thérapeutique s’est créé avec les membres de l’équipe soignante et les évaluations ont montré des traits névrotiques à savoir d’immaturité, de l’égocentrisme, et une avidité affective. Elle a accepté de voir ses parents contrairement à sa sœur, qui malgré une diminution de la composante sthénique demeure délirante et garde un jugement perturbé et une désorganisation comportementale. Au bout de cinq semaines une rémission thérapeutique est obtenue pour la patiente K à savoir une critique partielle du délire et diminution des symptômes dissociatifs, la patiente garde un émoussement de l’affectivité. L’évaluation à trois mois objective chez H des traits de personnalité pathologiques, elle remplit à la fois les critères DSM IV de la personnalité histrionique et dépendante.

## Discussion

Devant le contexte de relation étroite et fusionnelle, de totale identification avec un contenu psychotique et un comportement similaire nous avons posé le diagnostic de trouble psychotique partagé remplissant les critères DSM IV. Le sujet inducteur est dans notre cas la patiente K. le diagnostic retenu chez K est basé sur la présence de troubles de jugement et d’éléments dissociatifs et déficitaires. Un trouble délirant, vu la survenue tardive et la persécution en premier plan, a été évoqué mais l’évolution ultérieure a permis de l’éliminer sachant que ce diagnostic est le plus retrouvé en cas de psychose partagée. Un des diagnostics différentiels à l’induction d’une psychose au sein du couple est la survenue simultanée de troubles psychotiques dans la fratrie ou une prédisposition génétique jouerait un rôle important. Mais la réversibilité des symptômes chez la patiente H, juste après l’hospitalisation, écarterait une psychose chronique. Le diagnostic n’une bouffé délirante reste probable mais là aussi la rémission rapide et l’absence de symptômes hallucinatoire n’est pas en faveur. Il s’agit d’un cas intrafamilial entre une sœur plus âgée ayant une certaine emprise morale sur sa cadette, une situation fréquente dans les cas rapportés. La première observation clinique marquante fut le degré d’identification entre les deux sœurs, l’une est devenu l’écho de l’autre reproduisant non seulement le même délire mais aussi les mêmes attitudes toutes les deux ont poussé à l’extrême leur repli et leur absence d’hygiène et la perte marqué d’élan vital. Durant la mise en observation des patientes nous avons relevé le caractère étrange de la relation entre les deux sœurs comme le décrit Lacan au sujet du célèbre cas Papin: *"Il semble qu'entre elles, les deux sœurs ne pouvaient même prendre la distance qu'il faut pour se meurtrir. Vraies âmes siamoises, elles forment un monde à jamais clos"* et en parlant de leurs dépositions trop similaires *« on croit lire double… ».* En effet notre cas illustre bien ce confinement qui annule la distance et crée un système reclus rejetant toute intervention extérieure. Ainsi, durant le séjour à l’hôpital, H. a tenté par tous les moyens de voir K. de s’en approcher allant jusqu'à la menace suicidaire, et durant les entrevues les sœurs demeuraient accolées dénotant du caractère transférentiel de la relation au sein du couple délirant [6]: *« Je crois bien que dans une autre vie, je devrais être le mari de ma sœur »* disait la sœur Papin. Notre cas apporte un élément important qui est la question du rôle que peuvent jouer les traits de personnalité du patient secondaire dans la facilitation de la transmission du délire ou de la mise en place d’une relation intime et fusionnelle quasi-constante dans les cas de la littérature. Il revêt également un intérêt particulier de part l’extension du partage à d’autres symptômes, de nature schizophrénique non délirants. Il est vraisemblable que cette pathologie réponde à une mutifactorialité incluant des facteurs psychologiques, sociologiques culturelles mais aussi génétiques. Ces derniers ne peuvent être écartés dans les cas intrafamiliaux les plus fréquents, et la question du partage d’une prédisposition génétique- largement documentée notamment en matière de schizophrénie- se pose dans la FD et particulièrement en cas de folie simultanée qui classiquement désigne deux individus préalablement délirants qui développent et partagent un contenu délirant en collaboration. La survenue en intra fratrie est fréquente et plusieurs cas surviennent chez des jumeaux monozygotes comme en atteste une série de 16 cas rapporté par White [9]. Néanmoins il demeure rare que même chez des schizophrènes monozygotes qu’il y ait partage du même système délirant ce qui suggère que la similarité génétique seule ne permet pas d’expliquer le trouble. Lasègue posa la question fondamentale de la prédisposition à recevoir et à adhérer à un contenu délirant et pose des conditions nécessaires à cette transmission dont 3 semblent fondamentaux [8]: l’emprise et la supériorité morale du sujet inducteur; une relation étroite, fermée au monde extérieur; un délire vraisemblable, non bizarre.

Il met l’accent également sur la fonction du délire, et parle d’intérêt particulier du patient. Ces éléments d’observation traversent les discussions physiopathologiques actuelles notamment la relation en milieu clos qui réalise un isolement à deux où une proximité extrême tend vers la disparition réalisée du sujet, ceci traduisant vraisemblablement un souhait d’être soulagé de sa propre division, c’est-à-dire de s’abolir comme tel [10]. Quand à l’isolement, élément constant dont le rôle parait axial, il est légitime de se demander s’il n’est pas la conséquence de cette situation clinique plutôt que d’être considéré comme facteur étiologique. Au delà de la proximité de la relation, ca nature même interroge, elle est considérée comme transférentielle par Lacan et certains auteurs parlent même d’homosexualité latente [11]. Le rôle de la suggestibilité en sein d’une relation transférentielle reste une piste psychopathologique intéressante. Un model empirique de FD utilisant l’hypnose a montré que chez des patients fortement hypnotisables des idées délirantes ont été transmises expérimentalement [12], et il est légitime de se demander si ce n’est pas une condition nécessaire pour opérer toute transmission délirante. Nous discutons également le degré de parenté avec un certain nombre d’autres phénomènes psychiques regroupées sous le nom de folies contagieuses. Des cas rapportés de délire à trois ou à plusieurs aux croyances délirantes de la foule, en passant par des cas anecdotiques d’épidémies de danse ou de rire; aucune limite nette n’est établie. Déjà pour Clérambault les délires à deux, les délires fraternels, et les folies de quartier et épidémiques se mélangent sans trop de distinction avec comme point commun axial cette contagion du symptôme qui semble plus décisive que la nature du symptôme. Celui-ci varie volontiers selon le lieu et l’époque: une revue de la littérature réalisée par Sirois entre 1872 et 1972 considèrent que les mouvements anormaux étaient les manifestations les plus fréquemment partagées, devant les évanouissements et les dysesthésies pseudo-neurologiques, L’hystérie collective revêtirait à cette époque une clinique similaire à l’hystérie commune [13]. Avec le temps les épidémies de possession et d’hystéro-démonopathie des couvents ont laissé place à des craintes sanitaires concernant l’environnement et sa pollution potentielle, la possibilité d’infections ou le risque d’irradiation. Cette variabilité temporo spatiale du symptôme partagé semble fortement médiée par les données socioculturelles et englobe aussi bien des éléments psychotiques (délires, hallucinations) que d’autres manifestations psychiatriques. Une variabilité qui demeure valable dans la psychose partagée où des cas de folie à deux sur trouble bipolaire ou trouble dissociatif sont rapportées [14, 15]. Par ailleurs, ce rôle majeur de la culture dans la psychopathologie est très présent dans nos sociétés traditionnelles et largement retrouvé en clinique sous forme de délires de nature névrotique ou non psychotique [16-18]. Si cette notion semble désuète est dépassée en occident, elle demeure d’actualité dans le contexte de notre pratique. En effet, nous recevons beaucoup cliniques souvent rapidement résolutifs (surtout après hospitalisation) difficile à départager du délire psychotique, mais où le délire semble revêtir un sens avec notion de bénéfice et survient le plus souvent chez une patiente de sexe féminin avec des traits de personnalité histrionique. A la lumière de ces données et au delà d’une approche purement descriptive nous posons La question suivante: Le délire à deux est-il un délire d’origine névrotique? S’agit-il d’un modèle de délire non psychotique survenant sur un terrain particulier et répondant à une fonction? Outre les éléments sus-décrits qui vont dans le sens de notre supposition, notre argumentaire soulève aussi le parallèle possible entre traits de personnalité pathologique et les données de la littérature sur le trouble psychotique partagé, la suggestibilité, l’immaturité, la dépendance et l’avidité affective pourraient être des éléments clés dans le mode relationnel particulier caractérisant la quasi-totalité des cas de folie à deux rapportés. Plus encore, des données statistiques retrouvent plus de troubles de personnalité (personnalité passive) ainsi que plus d’événements de vie chez les sujets secondaires [3]. Mais alors quel est le sens de ce partage et quelle fonction joue le symptôme psychotique dans ce cas? Cette notion de bénéfice si inhérente au symptôme hystérique appelle à une approche fonctionnelle de l’activité délirante qui tient compte des enjeux subjectifs du délire, de ses éventuelles fonctions d’aménagement et de ses potentialités résolutives.

## Conclusion

Le peu de cas de folie à deux rapportés dans la littérature rend difficile une approche empirique d’où l’intérêt d’étude des cas qui fournissent un certain nombre de caractéristiques qu’on retrouve souvent quelque soit le milieu culturel. Nous avons tenté à travers ce cas de proposer des éléments de réflexion sur la psychopathologie du trouble; ceci dit il n’y a pas encore de modèle explicatif fiable et des questions restent ouvertes, par exemple la rareté de la pathologie malgré le nombre important de patients porteurs de symptomatologie délirante vivant en communauté, cette même question se pose dans les cas de fratries à deux ou plus de patients psychotiques mais qui pour la plupart ne partagent que rarement le même contenu délirant.

## Conflits d’intérêts

Les auteurs ne déclarent aucun conflit d'intérêts.

## Remerciements

Je remercie mes deux patientes et leur famille d’avoir accepté de publier leur cas. Je remercie également tous mes collègues et surtout le corps enseignant du Service de Psychiatrie CHU de Fès.
